# Supplementary material for: Physicochemical Stability of a Novel Tacrolimus Ophthalmic Formulation for the Treatment of Ophthalmic Inflammatory Diseases
Source: Pharmaceutics. 2022 Jan 4;14(1):118. doi: 10.3390/pharmaceutics14010118 (PMC8778879; doi:10.3390/pharmaceutics14010118)
Supplement: Supplementary file 1 [file pharmaceutics-14-00118-s001.zip › pharmaceutics-1518381SI1 final.pdf]

pharmaceutics

Supplementary Materials: Physicochemical Stability of a Novel Tacrolimus Ophthalmic Formulation for the Treatment of Ophthalmic Inflammatory Diseases

Marion Barrieu, Philip Chennell, Mouloud Yessaad, Yassine Bouattour, Mathieu Wasiak, Mireille Jouannet, Yoann Le Basle and Valérie Sautou

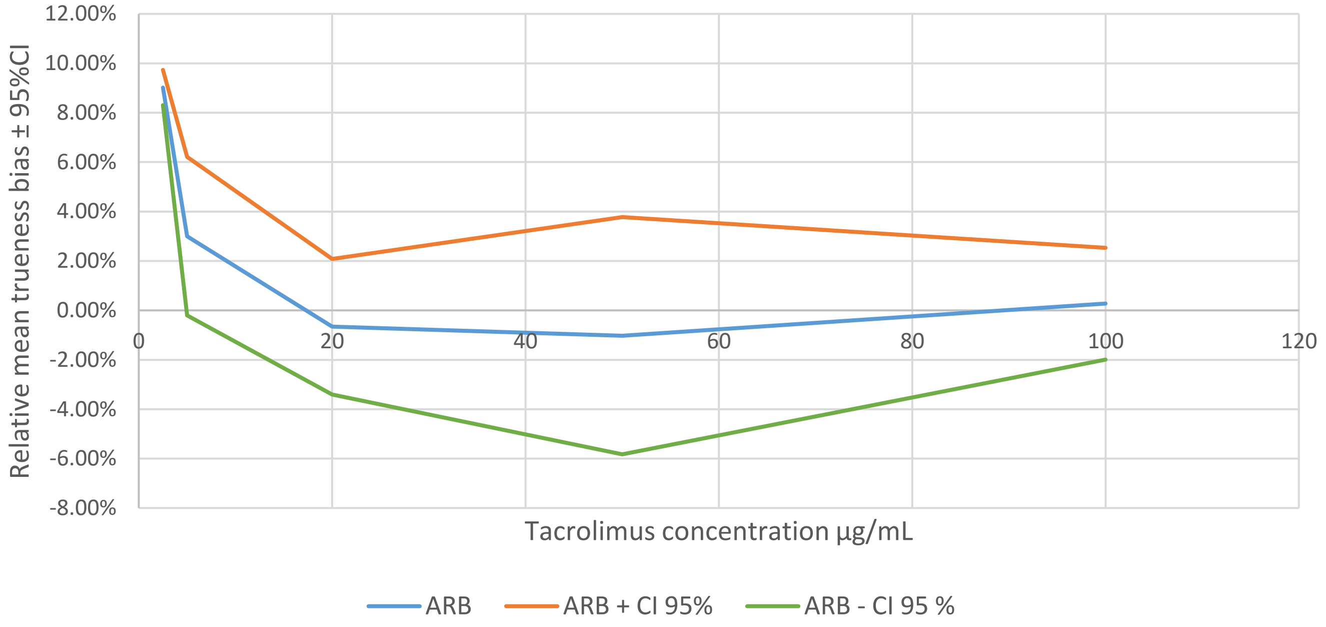

Figure S1. Accuracy profile of tacrolmus validation. ARB: Average relative trueness bias. CI: confidence interval.

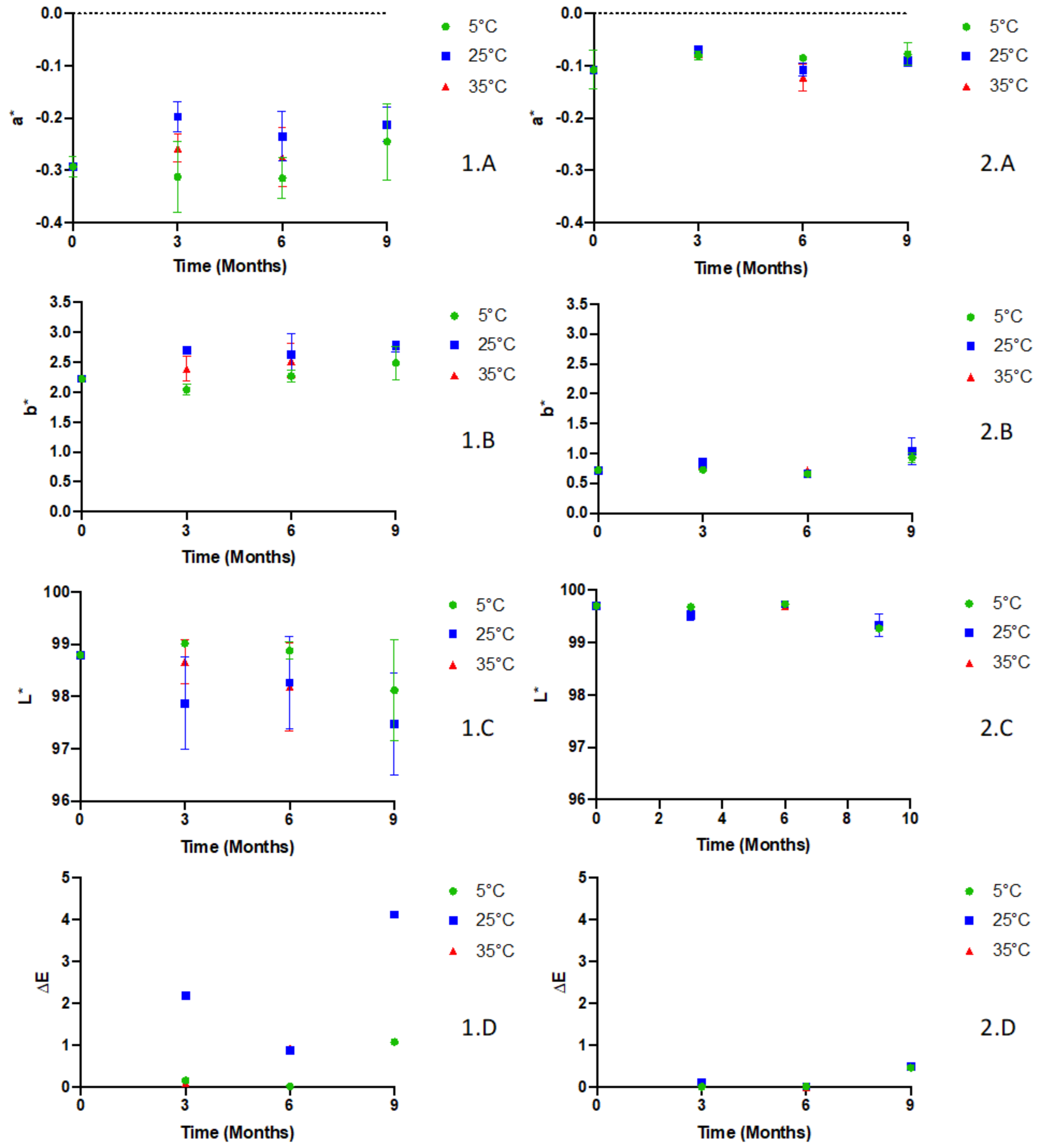

Figure S2. Evolution of chromaticity and luminance for the 1 mg/mL (1) and the 0.2 mg/mL formulation (2) with a* (A), b* (B), L*(C) and ΔE (D) during the 9 month stability study.

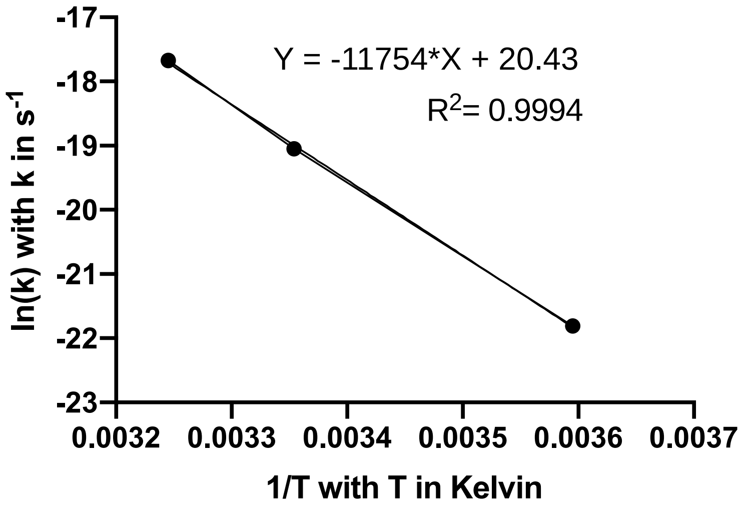

Figure S3. Applicability of the Arrhenius law.

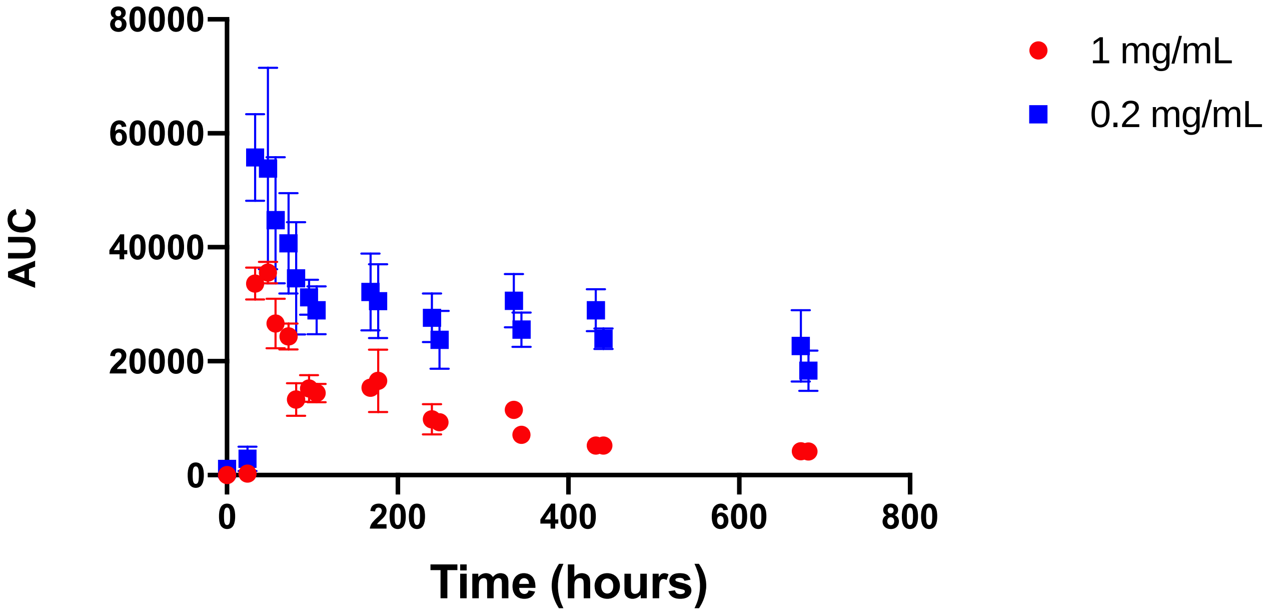

Figure S4. Evolution of the AUC of the leachable compound during the in-use assay at 5°C.

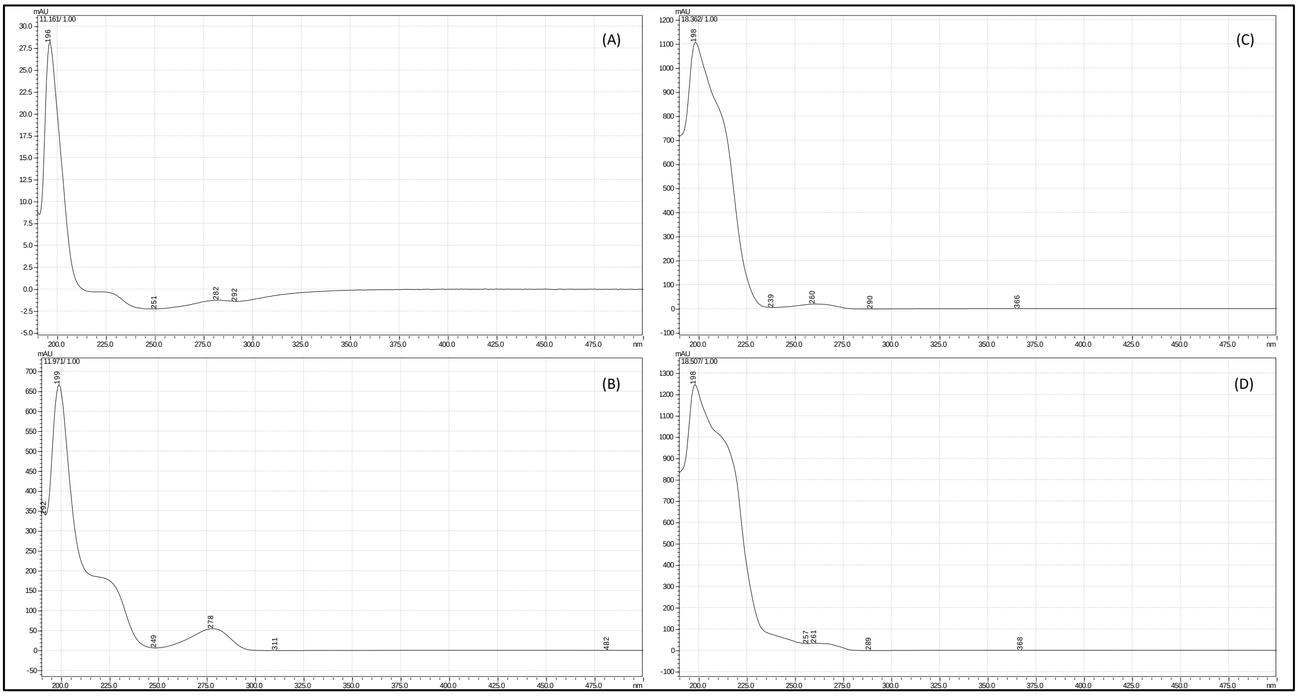

Figure S5. Comparison of the spectra UV of the leachable compound found in the in-use assay for the 1 mg/mL formulation after the delivery of the third drop (A) with the spectra UV of 2,4-Di-tert-butylphenol (B), 1,3-Di-tert-butylbenzene (C) and 1,4-Di-tert-butylbenzene (D).
